# Consultations and antibiotic treatment for urinary tract infections in Norwegian primary care 2006–2015, a registry-based study

**DOI:** 10.1186/s12875-021-01470-4

**Published:** 2021-06-24

**Authors:** Lars Emil Aga Haugom, Sabine Ruths, Knut Erik Emberland, Knut Eirik Ringheim Eliassen, Guri Rortveit, Knut-Arne Wensaas

**Affiliations:** 1 Research Unit for General Practice, NORCE Norwegian Research Centre, Postboks 22 Nygårdstangen, 5838 Bergen, Norway; 2 Department of Global Public Health and Primary Care, University of Bergen, Bergen, Norway

**Keywords:** Urinary tract infections, Cystitis, Pyelonephritis, Antibiotics, Primary care, General practice, After hours care

## Abstract

**Background:**

Extensive use of antibiotics and the resulting emergence of antimicrobial resistance is a major health concern globally. In Norway, 82% of antibiotics is prescribed in primary care and one in four prescriptions are issued for the treatment of urinary tract infections (UTI). The aim of this study was to investigate time trends in antibiotic treatment following a consultation for UTI in primary care.

**Methods:**

For the period 2006–2015 we linked data from the Norwegian Registry for Control and Payment of Health Reimbursements on all patient consultations for cystitis and pyelonephritis in general practice and out-of-hours (OOH) services, and data from the Norwegian Prescription Database on all dispensed prescriptions of antibiotics.

**Results:**

Altogether 2,426,643 consultations by attendance for UTI took place in the study period, of these 94.5% for cystitis and 5.5% for pyelonephritis. Of all UTI consultations, 79.4% were conducted in general practice and 20.6% in OOH services. From 2006 to 2015, annual numbers of cystitis and pyelonephritis consultations increased by 33.9 and 14.0%, respectively. The proportion of UTI consultations resulting in an antibiotic prescription increased from 36.6 to 65.7% for cystitis, and from 35.3 to 50.7% for pyelonephritis. These observed changes occurred gradually over the years. Cystitis was mainly treated with pivmecillinam (53.9%), followed by trimethoprim (20.8%). For pyelonephritis, pivmecillinam was most frequently used (43.0%), followed by ciprofloxacin (20.5%) and sulfamethoxazole-trimethoprim (16.3%). For cystitis, the use of pivmecillinam increased the most during the study period (from 46.1 to 56.6%), and for pyelonephritis, the use of sulfamethoxazole-trimethoprim (from 11.4 to 25.5%) followed by ciprofloxacin (from 18.2 to 23.1%).

**Conclusions:**

During the 10-year study period there was a considerable increase in the proportion of UTI consultations resulting in antibiotic treatment. Cystitis was most often treated with pivmecillinam, and this proportion increased during the study period. Treatment of pyelonephritis was characterized by more use of broader-spectrum antibiotics, use of both sulfamethoxazole-trimethoprim and ciprofloxacin increased during the study period. These trends, indicative of enduring changes in consultation and treatment patterns for UTIs, will have implications for future antibiotic stewardship measures and policy.

## Background

Extensive use of antibiotics and the increasing emergence of antimicrobial resistance is a major health concern globally [1–3]. In 2015, the World Health Organization launched a global action plan to improve antibiotic prescribing [4]. The Norwegian government aims at a 30% reduction in antibiotic use by 2020 compared to 2012-levels [5], and most of this must be achieved in primary care where about 82% of antibiotics is prescribed [6].

Urinary tract infections (UTIs) are the most common bacterial infections in females. Most women will experience an uncomplicated UTI during their lifetime, and one in five will have recurring infections [7]. Consequently, antibiotic stewardship in the management of UTI may be important to reduce overall use of antibiotics. The natural course of uncomplicated cystitis varies; studies indicate spontaneous resolution in 25–52% of patients within 1 week from symptom debut [8–10]. Antibiotic therapy significantly shortens the duration of symptoms compared to treatment with non-steroidal anti-inflammatory drugs [11–14] or placebo [8, 15, 16].

Whether to prescribe antibiotics is at the core of the clinical approach to cystitis. Withholding antibiotic therapy for cystitis has been associated with a small risk of progression to pyelonephritis [8–10, 17], but a meta-analysis of two randomized controlled trials revealed no statistically significant differences of antibiotics versus placebo in this regard [18]. In other European countries, most consultations in primary care for lower UTI results in antibiotic prescription, ranging from 60% in the Netherlands [19] to 74% in England [20], 84% in Sweden and 86% in Belgium [21]. Information on corresponding rates of antibiotic prescribing in Norway is lacking.

Guidelines for antibiotic treatment differ between countries depending on the resistance patterns for the most common pathogens. In Norway, national guidelines for antibiotic treatment in primary care were introduced in 2000 and are updated regularly [22]. Treatment recommendations for cystitis include nitrofurantoin, pivmecillinam or trimethoprim as a first-choice empirical antibiotic course. For the treatment of pyelonephritis, sulfamethoxazole-trimethoprim, pivmecillinam or ciprofloxacin are recommended as first-line agents.

The aim of this study was to investigate time trends in antibiotic treatment following consultations for UTI in primary care in Norway from 2006 to 2015.

## Methods

### Design and setting

This is an observational study based on nationwide data from primary care in Norway. Norway has a patient-list system entitling all residents to a regular general practitioner (GP). Most consultations in primary care are carried out in GP practices during opening hours, including daytime emergency consultations [23]. Municipal emergency medical services are organized as out-of-hours (OOH) services staffed by GPs on duty, or as 24-h service in the larger cities. During the study period the population of Norway increased by 11.3% from 4,640,219 individuals in 2006 to 5,165,802 in 2015 [24].

### Data sources

Information from two national registries for the period 2006–2015 was linked at the individual level, using the unique personal identity number (encrypted) assigned to all residents of Norway. The *Control and Payment of Health Reimbursement (KUHR)* Database contains data from all claims for fee-for-service from GPs and OOH services [25]. For each consultation, the claims contain GP- and patient-identifiers, date of consultation and one or more diagnoses according to the International Classification of Primary Care, 2nd version (ICPC-2). The *Norwegian Prescription Database (NorPD)* contains information on all prescription drugs dispensed from pharmacies to individual patients [26]. For each prescription, NorPD contains encrypted prescriber- and patient-identifiers, date of dispensing, and generic drug information (Anatomical Therapeutic Chemical (ATC) code). The NorPD does not include individual information on medication administered to hospitalized patients (~ 12,000 at any time) or nursing home residents (~ 40,000 at any time).

### Variables

We identified all consultations by attendance (not by telephone or e-consultation) to general practice and OOH services from January 2006 until December 2015 registered in KUHR with the diagnoses U70 pyelonephritis and U71 cystitis.

From NorPD we included all dispensed antibiotics for systemic use (ATC code J01). We considered antibiotics dispensed from pharmacies within 3 days following a UTI consultation to be linked to that consultation.

We created binary outcome variables for type of service (GP, OOH service), and dispensing of antibiotic for the treatment of UTI (yes, no). Type of antibiotics was categorized based on ATC codes.

We recoded patient age into the following categories: 0–9, 10–14, 15–19, 20–29, 30–39, 40–49, 50–59, 60–69, 70–79, and > 80 years.

### Statistical analysis

All descriptive analyses were conducted for cystitis and pyelonephritis separately. We investigate UTI consultations rather than UTI episodes. This implies that some consultations could possibly be for the same UTI episode, including scheduled follow-up visits after initiation of antibiotic treatment. We established the distribution of patient age and sex, and type of service for UTI consultations. We calculated the proportion of UTI consultations that were followed by antibiotic treatment and type of antibiotic dispensed. We explored time trends in the use of different antibiotics as treatment for UTI. Statistical significance was set at the 0.05 level. Analyses were performed using IBM SPSS Statistics for Windows, version 25.0 (IBM Corp., Armonk, NY, USA).

## Results

### Study population and consultations for UTI

During the 10-year study period, there were 2,426,643 consultations for UTI in primary care in Norway, out of a total of 140,199,637 consultations regardless of diagnosis (1.7%). Of all UTI consultations, 2,293,533 (94.5%) were for cystitis and 133,110 (5.5%) for pyelonephritis (Table 1). Most UTI consultations were conducted in general practice compared to OOH services, 1,927,615 (79.4%) vs. 499,028 (20,6%). This distribution was similar for cystitis consultations, 1,835,612 (80.0%) vs. 457,921 (20.0%), but the proportion of pyelonephritis consultations conducted in OOH services was relatively higher (41,107 (30.9%)) (Table 2).

**Table 1:** Consultations for cystitis and pyelonephritis in Norwegian primary care by patient sex and age, 2006-2015

Diagnosis	Age, years	All consultations, N	Males, %	Females, %
Cystitis	0–9	155,562	14.4	85.6
	10–19	149,708	5.5	94.5
	20–29	308,264	4.3	95.7
	30–39	245,884	6.8	93.2
	40–49	244,907	10.5	89.5
	50–59	260,282	15.0	85.0
	60–69	321,779	21.0	79.0
	70–79	320,175	23.1	76.9
	80+	286,972	24.3	75.7
	All ages	2,293,533	14.7	85.3
Pyelonephritis	0–9	8268	17.4	82.6
	10–19	10,474	7.0	93.0
	20–29	21,791	6.1	93.9
	30–39	17,474	13.1	86.9
	40–49	17,395	22.9	77.1
	50–59	18,016	30.9	69.1
	60–69	18,053	40.0	60.0
	70–79	12,530	38.8	61.2
	80+	9109	37.0	63.0
	All ages	133,110	23.2	76.8

All differences between sexes within age groups were statistically significant by Chi-square test

**Table 2:** Consultations and antibiotic treatment for cystitis and pyelonephritis in Norwegian primary care by consultation site, 2006-2015

Diagnosis	Year	General practice		Out of hours services	
		Consultations, N	Antibiotic treatment, %	Consultations, N	Antibiotic treatment, %
Cystitis	2006	149,982	34.1	37,289	47.0
	2007	143,031	37.6	38,646	49.6
	2008	164,119	41.0	43,488	52.1
	2009	164,163	44.3	46,341	54.8
	2010	187,718	48.2	49,937	57.0
	2011	195,750	51.5	51,183	60.0
	2012	200,527	54.8	51,010	62.7
	2013	208,143	58.4	49,909	64.9
	2014	213,910	61.5	47,679	67.4
	2015	208,269	64.9	42,439	69.7
	Sum	1,835,612	50.9	457,921	59.0
Pyelonephritis	2006	8213	28.0	3911	50.6
	2007	7033	28.8	3754	50.1
	2008	8682	32.3	4486	54.1
	2009	8534	35.2	4186	55.7
	2010	9177	36.2	4619	56.8
	2011	9374	38.9	4595	61.2
	2012	9823	40.7	4298	60.8
	2013	10,118	42.8	4161	61.9
	2014	10,504	44.4	3826	63.5
	2015	10,545	46.6	3271	64.0
	Sum	92,003	38.1	41,107	57.8

All differences in antibiotic prescription rate by consultation site and year were statistically significant by Chi-square test

**Fig. 1:**
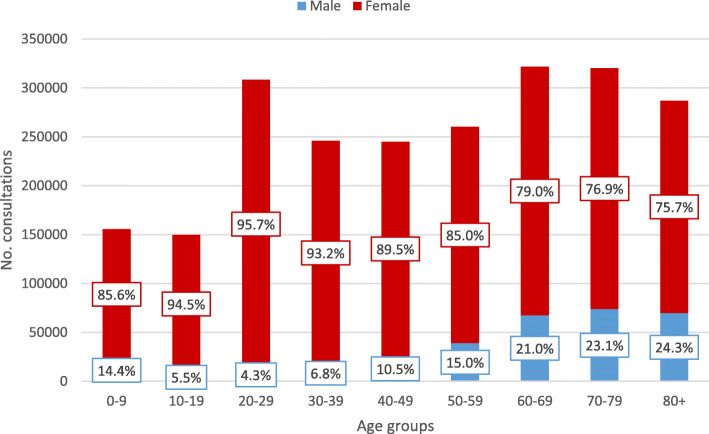
Consultations for cystitis by patient age and sex in Norwegian primary care, 2006–2015

**Fig. 2:**
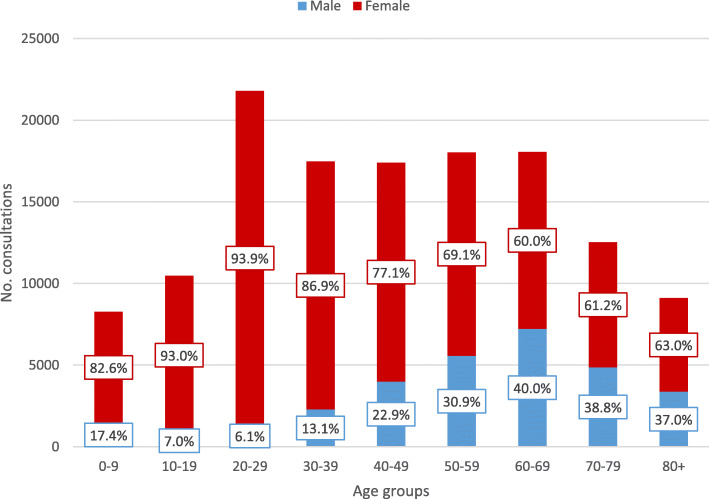
Consultations for pyelonephritis by patient age and sex in Norwegian primary care, 2006–2015

Female patients accounted for 85.3% of consultations for cystitis and 76.8% of consultations for pyelonephritis (Table 1, Figs. 1 and 2). Mean age of patients in cystitis consultations was 47.6 (SD 24.4, median 48.0, IQR 26.0–68.0) years for females and 60.1 (SD 23.5, median 66.0 IQR 49.0–78.0) years for males, whereas in pyelonephritis consultations mean age was 41.4 (SD 22.1, median 39.0 IQR 23.0–58.0) years for females and 56.0 (SD 20.7, median 60.0, IQR 45.0–70.0) years for males.

During the study period, the annual number of consultations for UTIs increased by 32.7% from 199,395 to 264,524. Consultations for cystitis increased by 33.9% (general practice, 38.9% and OOH services, 13.8%) and for pyelonephritis by 14.0% (general practice, 28.4% and OOH services, 16.4%) (Table 2).

### UTI and antibiotic treatment

**Fig. 3:**
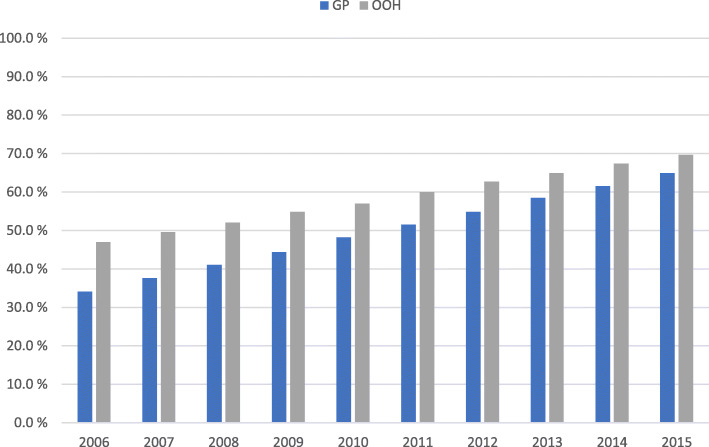
Proportion (%) of cystitis consultations resulting in antibiotics by consultation site, 2006-2015

**Fig. 4:**
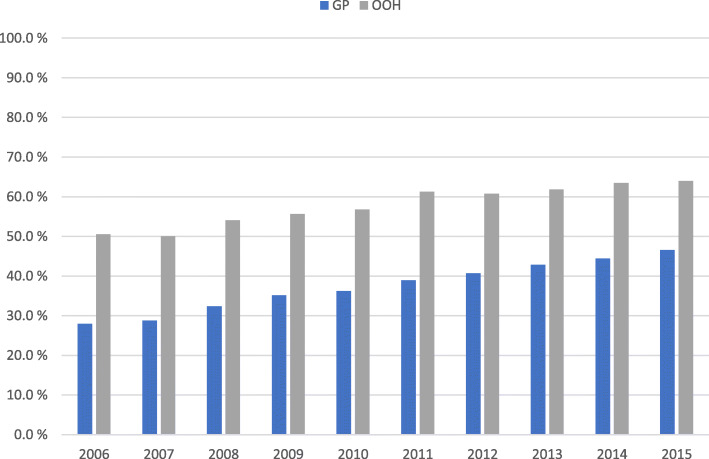
Proportion (%) of pyelonephritis consultations resulting in antibiotics by consultation site, 2006-2015

Altogether 1,263,543 of the 2,426,643 (52.1%) UTI consultations in primary care resulted in antibiotic treatment, and the proportion increased during the study period. Cystitis consultations resulting in antibiotic treatment increased from 36.6% in 2006 to 65.7% in 2015 in general practice, and from 47.0 to 69.7% in OOH services (Fig. 3). Pyelonephritis consultations resulting in treatment with antibiotics increased from 28.0 to 46.6% in general practice, and from 50.6 to 64.0% in OOH services (Fig. 4). For the study period as a whole consultations at OOH services more often resulted in antibiotics compared to daytime general practice for both cystitis (59.0% vs. 50.9%, *p* < 0.001) and pyelonephritis (57.8% vs. 38.1%, p < 0.001) (Table 2).

For both cystitis and pyelonephritis, a lower proportion of consultations resulted in antibiotics among the youngest and oldest patients (Table 3). Compared to female patients a lower proportion of consultations among male patients resulted in antibiotics for both cystitis (48.3% vs. 53.3%, p < 0.001) and pyelonephritis (38.4% vs. 45.9%, p < 0.001).

**Table 3:** Consultations and antibiotic treatment for UTIs in Norwegian primary care by age and sex, 2006-2015

Diagnosis	Age, years	Consultations both sexes		Males		Females	
		All	Consultations resulting in antibiotic treatment	All	Consultations resulting in antibiotic treatment	All	Consultations resulting in antibiotic treatment
		N	%	N	%	N	%
Cystitis	0–9	155,562	41.4	22,342	33.8	133,220	42.6
	10–19	149,708	54.7	8307	45.5	141,401	55.2
	20–29	308,264	54.7	13,202	49.0	295,062	55.0
	30–39	245,884	54.9	16,644	49.0	229,240	55.3
	40–49	244,907	57.3	25,674	51.7	219,233	57.9
	50–59	260,282	56.3	38,944	51.9	221,338	57.1
	60–69	321,779	54.0	67,578	51.9	254,201	54.5
	70–79	320,175	50.6	73,903	49.4	246,272	51.0
	80+	286,972	46.1	69,826	45.1	217,146	46.5
	Sum	2,293,533	52.5	336,420	48.3	1,957,113	53.3
Pyelonephritis	0–9	8268	40.9	1442	33.5	6826	42.5
	10–19	10,474	50.7	735	41.2	9739	51.4
	20–29	21,791	49.1	1327	41.6	20,464	49.6
	30–39	17,474	47.4	2295	43.4	15,179	48.0
	40–49	17,395	46.0	3986	42.5	13,409	47.0
	50–59	18,016	43.3	5573	40.1	12,443	44.7
	60–69	18,053	41.8	7225	39.0	10,828	43.6
	70–79	12,530	37.7	4864	36.1	7666	38.7
	80+	9109	33.4	3370	30.1	5739	35.4
	Sum	133,110	44.2	30,817	38.4	102,293	45.9

All differences between sexes within age groups in terms of antibiotic treatment were statistically significant by Chi-square test

### Type of antibiotics

We report the five most frequently used antibiotics for treatment of cystitis and pyelonephritis, which accounted for 95.0 and 91.8% of antibiotic dispenses for these conditions, respectively. For cystitis these were, in descending order, pivmecillinam, trimethoprim, nitrofurantoin, ciprofloxacin and sulfamethoxazole-trimethoprim. For pyelonephritis, the five most frequently used antibiotics in descending order were pivmecillinam, ciprofloxacin, sulfamethoxazole-trimethoprim, amoxicillin and trimethoprim (Table 4).

**Table 4:** The 5 most frequently prescribed antibiotics for cystitis and pyelonephritis by patient age, 2006–2015

Cystitis	Age	Total	Pivmecillinam	Trimethoprim	Nitrofurantoin	Ciprofloxacin	Sulfa-Trim^a^	Other
		N	%	%	%	%	%	%
	0–9	64,338	19.1	45.0	5.6	0.3	16.8	13.1
	10–19	81,836	61.6	22.5	4.8	2.7	4.4	4.1
	20–29	168,771	64.1	17.8	6.2	3.4	3.6	4.9
	30–39	134,920	61.1	18.4	7.1	4.5	4.0	4.9
	40–49	140,210	58.1	19.8	7.3	6.1	4.7	4.0
	50–59	146,576	55.3	19.3	8.3	7.7	5.3	4.1
	60–69	173,693	51.6	18.9	9.4	9.5	5.9	4.6
	70–79	162,037	49.5	19.6	10.4	10.1	5.8	4.6
	80+	132,382	47.8	20.5	10.6	10.4	5.7	5.0
	Sum	1,204,763	53.9	20.8	8.1	6.7	5.6	5.0
Pyelonephritis	Age	Total	Pivmecillinam	Ciprofloxacin	Sulfa-Trim^a^	Amoxicillin	Trimethoprim	Other
		N	%	%	%	%	%	%
	0–9	3389	30.9	1.0	27.6	12.7	9.4	18.3
	10–19	5330	50.8	14.0	15.9	7.2	4.4	7.3
	20–29	10,771	49.9	16.6	15.0	6.9	4.0	7.0
	30–39	8334	46.5	18.8	14.4	6.8	4.7	8.1
	40–49	8084	42.4	22.3	15.8	6.9	4.4	7.2
	50–59	7886	39.6	25.7	15.1	7.1	4.1	7.3
	60–69	7674	37.2	26.5	16.2	6.9	3.7	7.6
	70–79	4825	34.4	27.5	16.2	7.3	4.1	8.2
	80+	3138	37.9	23.4	14.7	7.6	4.9	8.6
	Sum	58,780	43.0	20.5	16.3	7.4	4.6	8.2

All differences in type of antibiotic per age-group statistically significant by Chi-square test

^a^ Sulfamethoxazole-trimethoprim

Cystitis was predominantly treated with pivmecillinam (53.9%), followed by trimethoprim (20.8%). Young children (0–9) with cystitis were mostly treated with trimethoprim (45.0%), pivmecillinam (19.1%) or sulfamethoxazole-trimethoprim (16.8%). For pyelonephritis, pivmecillinam was most frequently used (43.0%), followed by ciprofloxacin (20.5%) and sulfamethoxazole-trimethoprim (18.1%). The youngest and oldest patients were more often treated with broad-spectrum antibiotics for pyelonephritis; sulfamethoxazole-trimethoprim accounted for 27.6% of prescriptions for children 0–9 years old, and ciprofloxacin accounted for 27.5 and 20.5% of prescriptions for patients 70–79 and > 80 years (*p* < 0.001), respectively.

**Fig. 5:**
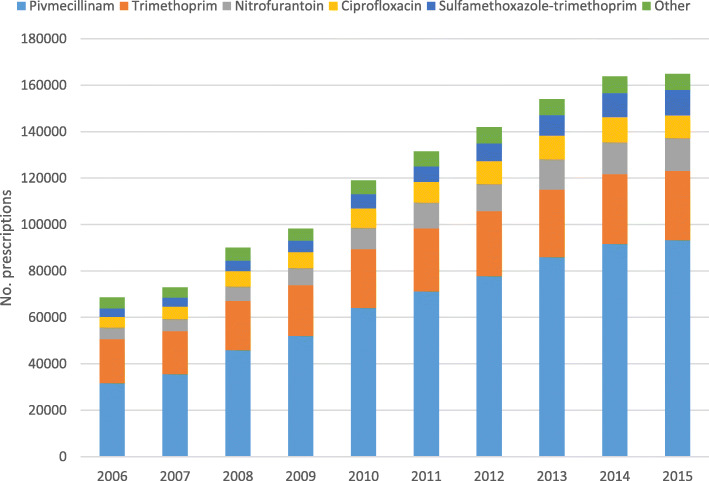
The 5 most frequently prescribed antibiotics for cystitis in Norwegian primary care by year, 2006–2015

**Fig. 6:**
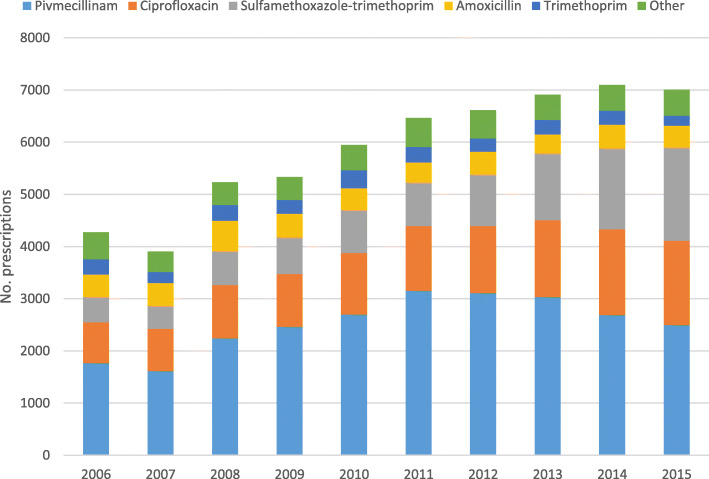
The 5 most frequently prescribed antibiotics for pyelonephritis in Norwegian primary care by year, 2006–2015

From 2006 through 2015 we observed gradual and consistent changes in the type of antibiotic prescribed. Pivmecillinam was the most frequently prescribed antibiotic for cystitis and the proportion increased from 46.1 to 56.6% (Fig. 5). For pyelonephritis, the use of sulfamethoxazole-trimethoprim increased from 11.4 to 25.5%, and ciprofloxacin from 18.2 to 23.1%, while the use of pivmecillinam decreased from 41.1 to 35.6% (Fig. 6).

## Discussion

### Summary

In this nationwide registry-based study, we found a 32.7% increase in UTI consultations in primary care in Norway from 2006 to 2015. The number of consultations for cystitis increased more than consultations for pyelonephritis. Further, the proportion of UTI consultations resulting in an antibiotic prescription increased from 36.6 to 65.7% for cystitis, and from 35.3 to 50.7% for pyelonephritis. The increase in number of consultations for cystitis, as well as the proportion of these consultations leading to antibiotic treatment, was greater in general practice compared to OOH services. Pivmecillinam was the most frequently used antibiotic for both cystitis and pyelonephritis, but there was a shift towards more use of broader-spectrum antibiotics like ciprofloxacin and combined sulfamethoxazole-trimethoprim in the treatment of pyelonephritis.

### Strengths and limitations

The main strength of this study is the use of complete registry data from the publicly funded primary care services in Norway. Linkage of data from two registries at the individual patient level provides a rich source of information. Use of registry data eliminates recall bias and considerably reduces selection bias.

Clear information regarding GP-diagnosed UTI is another strength, defined as a GP-consultation with the ICPC-2 code U71 cystitis or U70 pyelonephritis. However, the individual GPs decide the diagnosis in each consultation and the KUHR database contains no variables that will aid validation of UTI diagnoses. Differences in coding behavior may therefore challenge the internal validity. Potential misclassification would probably be related to the GP more than to the patient and results comparing sex, age groups and different antibiotics should be less influenced by this bias. Reimbursement claims from contacts by telephone or at the reception were not included in this study. This is a limitation as UTIs, especially cystitis, may be managed by such short contacts rather than by face-to-face consultation, even when antibiotics are prescribed. A recent study from Norwegian general practice showed that misclassification of diagnoses is higher for contacts via the reception only or by telephone than for consultations by attendance. Short contacts of this kind are frequent and only a small proportion will relate to UTIs. It is likely that including these contacts would give less accurate data [27]. E-consultations were introduced in Norwegian primary care at the end of the study period, in 2015, and are therefore not included in this study.

The NorPD contains complete data on all prescription drugs *dispensed*. Although we may have slightly underestimated *prescribed* antibiotics due to primary non-compliance, drug dispensing data is recognized as an acceptable proxy for drug use in in epidemiological studies [28]. Linkage of antibiotic dispensing to UTI diagnosis by time intervals of maximum 3 days supports our assumption regarding the reasons for prescribing.

### Interpretation/comparison with existing literature

During the study period the population of Norway increased by 11.3% [24]. In the same period, the number of cystitis consultations increased more (33.9%) than consultations for all causes (23.5%) [29]. This may suggest a trend towards more cystitis contacts being performed as consultation by attendance rather than by telephone or other indirect non-physical means. Face-to-face consultation allow for a physical examination, laboratory testing and a more thorough assessment of whether to treat with antibiotics. This might be a result of increased awareness of antibiotic stewardship, or possibly economic incentives. Fees and reimbursements are higher for consultations than for short contacts, but this was constant during the study period.

Another challenge is how to interpret our findings in the light that we review consultations due to UTI, not UTI-cases (which are possibly made up of multiple consultations). We observed that proportionally more UTI consultations resulted in antibiotics during the study period. This could be due to more antibiotic prescribing per UTI-case, or it could indicate fewer consultations per UTI-case.

We found that the proportion of cystitis consultations resulting in antibiotic treatment increased from 36.6% in 2006 to 65.7% in 2015. Paradoxically, overall use of antibiotics (all forms of contact, consultations, telephone etc.) typically associated with treatment of cystitis in Norwegian primary care (for instance pivmecillinam, trimethoprim and nitrofurantoin) decreased from 2010 to 2015 according to the NORM/NORM-Vet report [30]. Access to antibotic treatment in Norway is only possible by a prescribing physician.

We interpret these findings as indicative of a change in how cystitis is managed in primary care. A likely explanation is that short contacts for UTI most often resulted in antibiotic prescription, while the more unsure cases led to a face-to-face consultation without prescribing. As UTIs increasingly are managed by a consultation the proportion resulting in antibiotic prescription is higher for consultations, but lower for all sorts of contact combined. As coding of short contacts is less accurate than for consultations we conclude that reimbursement claims for short contacts will not provide data that can reliably answer this question [27].

Compared to other European countries with comparable healthcare systems the percentage of cystitis consultations resulting in antibiotic treatment in Norwegian primary care is low, even towards the end of our study period. A cohort study of GP-practices in Belgium, the Netherlands and Sweden with data collected for the year 2012 found that 87, 67, and 84% of cystitis consultations resulted in antibiotic treatment, respectively [21]. A Swedish cohort study in GP-practices, from 2014 to 16, found that 74% of women with cystitis were treated with antibiotics [31]. A household survey from the UK performed during 2014 reported 74% of cystitis consultations resulting in antibiotics [20]. A cohort study in GP-practices in Switzerland 2017–18 found that 92.4% of patients consulting for cystitis received antibiotics [32].

In our study the proportion of pyelonephritis consultations resulting in antibiotic treatment was low, but increased during the study period from 35.3% in 2006 to 50.7% in 2015. This apparently low prescription rate is likely to reflect that pyelonephritis is a more severe infection with a higher proportion of consultations leading to hospital admittance for definitive treatment – thus decreasing the proportion of consultations in our dataset that led to antibiotic treatment. We do not have access to data on hospitalization. Other cases of pyelonephritis may have been followed up several times by the GP, with prescription only once. An increase in the number of pyelonephritis consultations resulting in ambulatory antibiotic treatment towards the end of the study period suggests that either proportionally fewer cases of pyelonephritis were admitted to the hospital or that fewer subsequent control-consultations (without prescribing) were performed. Trends in other European countries regarding the proportion of pyelonephritis consultations resulting in antibiotic treatment is difficult to assess, in part due to the scarcity of population-based studies on the condition and the age of those studies that do exist [33, 34].

The proportion of UTI consultations resulting in antibiotic treatment was lower among male patients and the youngest and oldest age groups. Norwegian national guidelines define that cystitis in male is a “complicated infection” and recommend empirical antibiotic treatment [22]. In this study we review UTI consultations in primary care, not cases, and similar to pyelonephritis a lower rate of consultations resulting in antibiotics could therefore be due to a higher number of follow-up visits without prescribing or more often admittance to secondary care such as hospitals for definitive antibiotic treatment.

We found that pivmecillinam was most frequently used for both cystitis and pyelonephritis. The youngest (0-9y) and oldest (>70y) patients received more often broad-spectrum antibiotics for both cystitis and pyelonephritis. Contributing factors could be more vague symptoms [35] or a higher risk of severe infection in the case of therapy failure in these age-groups, thus calling for the use of more broad-spectrum antibiotics [36].

Ciprofloxacin use for UTIs in Norwegian primary care fell for cystitis (from 6.6% of antibiotics prescribed for the condition in 2006 to 5.9% in 2015) and increased for pyelonephritis (from 18.2% in 2006 to 23.1% in 2015). In a European context, there appears to be some variation in the use of fluoroquinolones for UTIs. Sweden, the Netherlands, and Switzerland have comparable low rates of fluoroquinolone use for cystitis at 3.0, 7.4 and 6.0% respectively [21, 32], whereas a study from Hungary found that 56.2% of cystitis cases were treated with a fluoroquinolone [37]. For pyelonephritis, a national registry study from Denmark with data for the period 2012–13 found that quinolones made up 19.7% of antibiotics prescribed [38].

### Implications for clinical care and research

The management of cystitis remains a prime candidate for antibiotic stewardship measures; especially as the condition is prevalent and potentially self-limiting.

A surprising finding in our study was that both the total number of UTI-consultations and the proportion of UTI-consultations leading to antibiotic treatment increased gradually during the study period. As discussed above, there are several factors that could contribute to this development; perhaps most important a possible enduring shift in consultation trends, where UTI increasingly is managed as face-to-face consultations. This introduces the possibility for individually targeted treatment for each patient with a potential of further reduction in antibiotic prescribing.

To support this, more knowledge is needed about non-antibiotic management, the risk of complications and proper safety netting, both through clinical trials and epidemiological studies on the course of UTI episodes.

## Data Availability

The data that support the findings of this study are available from the KUHR database and NorPD, but restrictions apply to the availability for these data, which were used under license for the current study, and so are not publicly available. Data are however available from the authors upon reasonable request and provided permission is granted from the KUHR database and NorPD.
